# Neonatal seizures as onset of Inborn Errors of Metabolism (IEMs): from diagnosis to treatment. A systematic review

**DOI:** 10.1007/s11011-021-00798-1

**Published:** 2021-08-17

**Authors:** Raffaele Falsaperla, Laura Sciuto, Luisa La Spina, Sarah Sciuto, Andrea D. Praticò, Martino Ruggieri

**Affiliations:** 1 Unit of Pediatrics and Pediatric Emergency, University Hospital Policlinico “Rodolico-San Marco”, Catania, Italy; 2 Unit of Neonatal Intensive Care and Neonatology, University Hospital Policlinico “Rodolico-San Marco”, Catania, Italy; 3 Pediatrics Postgraduate Residency Program, Section of Pediatrics and Child Neuropsychiatry, Department of Clinical and Experimental Medicine, University of Catania, Via S. Sofia 78, 95123 Catania, Italy; 4 Regional Reference Center for the Treatment and Control of Congenital Metabolic Diseases of Childhood, University Hospital Policlinico “Rodolico-San Marco”, Catania, Italy; 5 Unit of Rare Diseases of the Nervous System in Childhood, Department of Clinical and Experimental Medicine, Section of Pediatrics and Child Neuropsychiatry, University of Catania, Catania, Italy

**Keywords:** Neonatal seizures, Inborn errors of metabolism, Seizures, Neonate

## Abstract

Neonatal seizures (NS) occur in the first 28 days of life; they represent an important emergency that requires a rapid diagnostic work-up to start a prompt therapy. The most common causes of NS include: intraventricular haemorrhage, hypoxic-ischemic encephalopathy, hypoglycemia, electrolyte imbalance, neonatal stroke or central nervous system infection. Nevertheless, an Inborn Error of Metabolism (IEM) should be suspected in case of NS especially if these are resistant to common antiseizure drugs (ASDs) and with metabolic decompensation. Nowadays, Expanded Newborn Screening (ENS) has changed the natural history of some IEMs allowing a rapid diagnosis and a prompt onset of specific therapy; nevertheless, not all IEMs are detected by such screening (e.g. Molybdenum-Cofactor Deficiency, Hypophosphatasia, GLUT1-Deficiency Syndrome) and for this reason neonatologists have to screen for these diseases in the diagnostic work-up of NS. For IEMs, there are not specific semiology of seizures and EEG patterns. Herein, we report a systematic review on those IEMs that lead to NS and epilepsy in the neonatal period, studying only those IEMs not included in the ENS with tandem mass, suggesting clinical, biochemical features, and diagnostic work-up. Remarkably, we have observed a worse neurological outcome in infants undergoing only a treatment with common AED for their seizures, in comparison to those primarily treated with specific anti-convulsant treatment for the underlying metabolic disease (e.g.Ketogenic Diet, B6 vitamin). For this reason, we underline the importance of an early diagnosis in order to promptly intervene with a targeted treatment without waiting for drug resistance to arise.

## Introduction

Neonatal seizures (NS) occur in the first 28 days of life and represent a clinical emergency that could need Intensive Neonatal cares. Moreover, in the neonatal period, seizures could represent the first clinical sign of a significant neurologic dysfunction, which underlies other disease (Loman et al. 2014)(Shellhaas 2021).

The most common causes of seizures in the neonatal period include: intraventricular haemorrhage in preterm newborns, and hypoxic-ischemic encephalopathy in those at term; other common causes include hypoglycaemia, electrolyte imbalance (hypomagnesemia, hyponatremia, hypocalcaemia), neonatal stroke or central nervous system infections (Chan et al. 2010). Inborn Errors of Metabolism (IEMs) are an uncommon cause of NS. In this regard, Loman et al. in their study on term newborns found that IEMs caused NS in 5/221 (2,3%) of newborns (Loman et al. 2014). Reversely, epileptic seizures are a common feature in several IEMs; indeed, 85% of IEMs displays predominantly neurological manifestations (Choudhry et al. 2013).

Inborn Errors of Metabolism (IEMs) are a group of congenital disorders due to a genetic defect that leads to a metabolic derangement in a biochemical pathway, with a secondary involvement of many organs including brain. Even if individually rare, they are collectively numerous and can be divided in three groups based on a pathophysiological point of view (Plana 2019). Clinically heterogeneous, NS related to IEMs can be clonic, tonic, spasms, and myoclonic, usually associated with transient eye deviations, nystagmus, blinking, mouthing, boxing, bicycling movements, and apnea (Parisi et al. 2015). Electrographic discharges are often detected even though there is no specific EEG pattern (Papetti et al. 2013). Thus, diagnosis of IEM in case of NS needs a high index of suspicion from neonatologists (Ficicioglu and Bearden 2011).

Nowadays, Expanded Newborn Screening (ENS) has changed the natural history of some IEMs allowing a rapid diagnosis and prompt onset of a specific therapy (Jaume and Plecko 2015). Unfortunately, not all IEMs can be included in the Program of screening and some present before screening tests results are available. In these cases, seizures of metabolic origin are suspected when drug resistance appears, defined as the failure to respond to at least two antiseizure drugs (Berg et al. 2001). For this reason, neonatologists and paediatricians should include IEMs not studied in the standard ENS in their diagnostic flow-chart, above all when a newborn develops seizures. They should consider prompt diagnostic investigations in order to start targeted therapies (e.g. Ketogenic diet) (Ismayilova et al. 2018) as soon as possible. Moreover, it should be noticed that for those IEMs for which therapy is not available, the diagnosis is still important for genetic counselling and recurrence risk (Brimble and Ruzhnikov 2020).

Herein, we provided a systematic review of the literature on studies regarding those IEMs that are not screened with ENS, and/or Tandem Mass and that cause NS and epilepsy in neonatal age. We aimed to focus on the importance to start early in-depth diagnostic investigations, avoiding the waste of time, which is related to the development of drug resistance. We aimed to show that time of diagnosis and onset of a specific therapy is decisive of the neurocognitive outcome of these infants.

## Materials and methods

### Search strategy

The present systematic review was conducted following the general principles established by Preferred Reporting Items for Systematic Reviews and Meta-Analyses (PRISMA) and the Institute of Medicine Standards for Systematic Review (Liberati et al. 2009). We perfermed the search of a five-year period, from 2016 to 2021, as in the last five years expanded newborn screening has become extensively available (and used) in most of the Western countries. We collected data from PubMed/Medline and Scopus database using the following MeSH terms: “Inborn error of metabolism AND neonatal seizures”.

We included all the studies describing patients with neonatal (i.e. 0–28 days) seizures caused by IEMS and/or describing neonatal seizures demonstrated by EEG findings. All the studies had to be written in English language and conducted in humans.

Studies published before 2016 and describing patients older than 28 days were excluded, as well as those reporting patients affected by diseases diagnosed with Tandem Mass. We excluded also reviews, meta-analysis and descriptive studies not including patients.

### Description

**Fig. 1:**
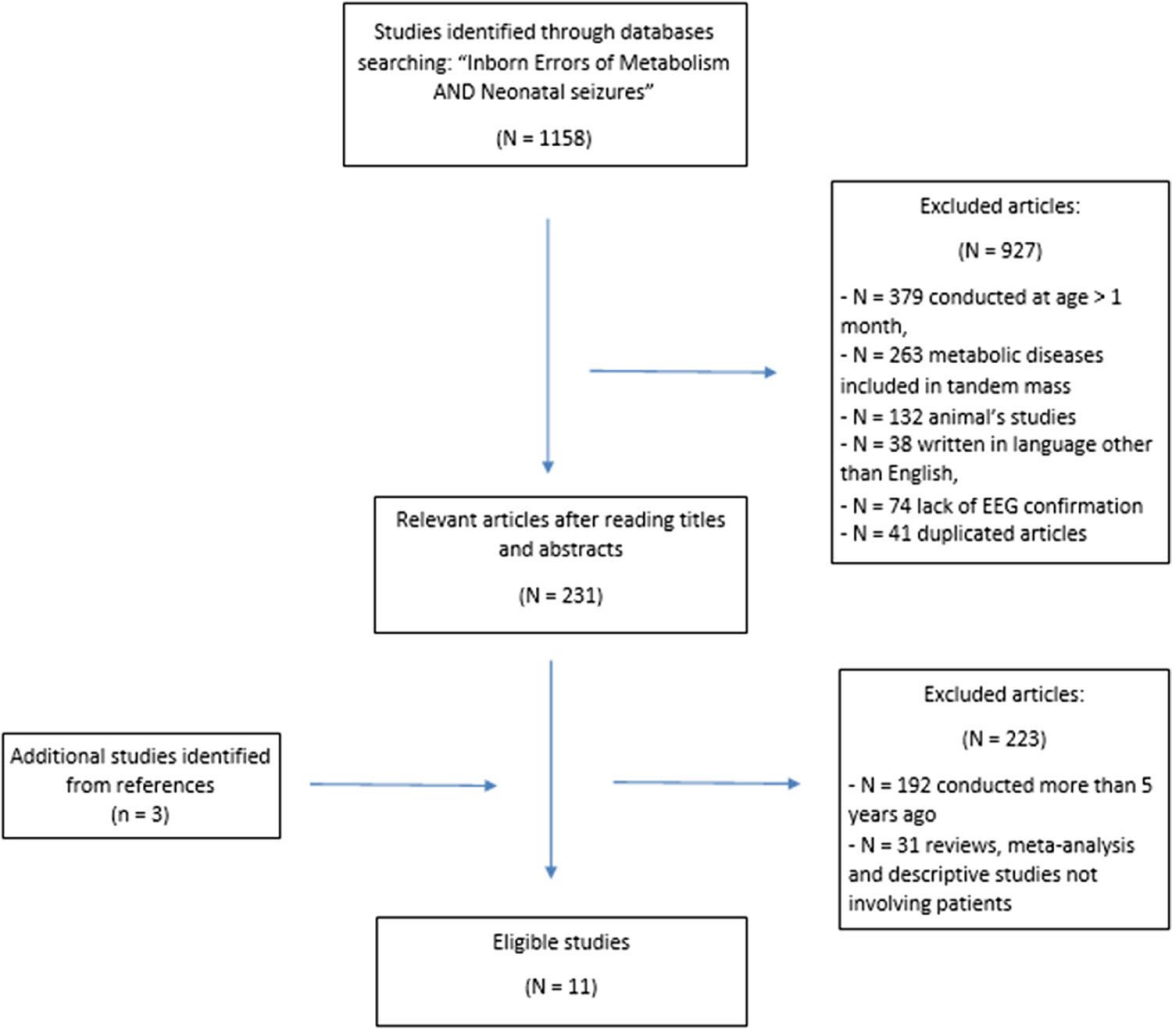
Flow chart of the search and study selection process

Our PUBMED search for “Inborn Errors of Metabolism and neonatal seizures” identified 1158 articles. After reviewing the titles and abstracts of these articles, we reviewed the full texts of 231 articles and rejected studies matching the exclusion criteria. Eight of these fulfilled the criteria for inclusion in the review. Moreover, analysing the references of the eight selected articles, we added three other relevant articles to the review. For this reason, a total of 11 articles were finally included in our review (Fig. 1).

### Methodological quality

Our systematic review was assessed using the “Assessing the Methodological Quality of Systematic Reviews 2”( AMSTAR 2) criteria (Lu et al. 2020). According to AMSTAR 2 score, “high quality review” result was obtained for this review (score 8).

## Results

Given the lack of robust evidence and the rarity of the topic discussed, only case reports were collected from the scientific literature and therefore included in our systematic review.

A total of 13 cases of infants with neonatal seizures caused by Inborn Errors of Metabolism have been collected from literature data during the period of 2016 to 2021 in 11 studies (Table 1).

**Table 1:** Relationship between treatment and neurological outcome

AUTHORS	STUDIES	IEOM	CLINICAL FEATURES OF SIZURES	EEG	FIRST TREATMENT	RESOLUTION OF SEIZURES	OTHER TREATMENTS	AGE AT ONSET	NEUROLOGICAL OUTCOME
Hande et al.(Gazeteci-Tekin et al. 2019)	case report	PDE	infantile spasms, status epilepticus with generalized seizures	INTERICTAL EEG: burst suppression pattern	Common AEDs	yes	B6 vitamin 30 mg/kg/d	5 month of life	severe hypotonia, severe intellectual disability and motor delay
Cirillo et al.(Cirillo et al. 2015)	case report	PDE	no suppressible rhythmic movements of her extremities with associated eye deviation and oxygen desaturation (myoclonic-tonic and brief tonic seizures)	CONTINUOUS VIDEO EEG: excessive multifocal sharp wave discharges and discontinuity for the stated post-conceptual age	pyridoxine (100 mg once daily) + phenobarbital	no	B6 vitamin 150 mg once daily	first week of life	normal
By H. M.J. Slot1 et al.(Slot et al. 1993)	case report	MCD	tonic–clonic seizures	INTERICTAL EEG: intermittent paroxysms of sharp waves over the left temporal region, followed by burst suppressions of four to six seconds	Diazepam	yes	/	/	progressive deterioration
		MCD	alternating tonic and tonic–clonic seizures	INTERICTAL EEG: multifocal epileptic phenomena, predominantly over the right hemisphere	Common AEDs	no	/	/	severe neurological abnormalities
Meyer et al.(Slot et al. 1993)	case report	CLN 10	myoclonic seizures	INTERICTAL EEG: burst suppression pattern	Common AEDs	no	/	/	severe neurological abnormalities
		CLN 10	myoclonic seizures	INTERICTAL EEG: burst suppression pattern	Common AEDs	yes	/	/	severe neurological abnormalities
Ishiguro et al.(Ishiguro et al. 2019)	case report	HYPOPH	subclinical (maybe masked by sedative drugs and muscle relaxant)	VIEDO EEG: burst suppression pattern electrical seizure cluster	pyridoxine hydrochloride (30 mg/kg) iv and ERT (asfotase alfa)	yes	/	/	normal
Tarek et al.(Belal et al. 2018)	case report	GLUT1-DS	apnea, desaturations and eye rolling with "arm bicycling"- like movements	VIDEO EEG: multiple seizure episodes	Common AEDs	no	Ketogenic diet	10 day of life	normal
Arnold et al.(Arnold et al. 1993)	case report	MCD	tonic seizures, followed by apnea and shock	INTERICTAL EEG: multifocal epileptiform discharges	Common AEDs	yes	/	/	progressive deterioration
Baumgartner-Sigl et al.(Baumgartner-Sigl et al. 2007)	case report	HYPOPH	multifocal myoclonic jerks and tonic seizures	INTERICTAL EEG: continuous burst suppression pattern,	Common AEDs	no	Intravenous PN (Bena-don®), 100 mg (60 mg/kg/day)	12 day of life	normal
Çolak et al.(Çolak et al. 2017)	case report	GLUT1-DS	hiccups and mandibular sign seizures	INTERICTAL EEG: generalised epileptic deterioration	Common AEDs	no	ketogenic diet	3 month of life	normal
Fukazawa et al.(Fukazawa et al. 2018)	case report	HYPOPH	brief tonic convulsions combined with the setting sun sign	INTERICTAL EEG: multifocal epileptic discharges	i.v. B6 vitamin (10 mg/kg/day pyridoxal phosphate)	yes	i.v. pyridoxal phosphate dose was increased (to 40 mg/kg/day)	1 month of life	psychomotor delay
Demirbilek et al.(Demirbilek et al. 2011)	case report	HYPOPH	generalized tonic–clonic seizures	INTERICTAL EEG: epileptic activity in the left frontal area	Common AEDs	yes	Pyridoxine	4 month of life	n.a

Pyridoxine-dependent epilepsy (PDE); Molybdenum-Cofactor Deficiency (MCD); Infantile Neuronal Ceroid Lipofuscinosis (CLN10); hypophosphatasia (HYPOPH); GLUT1-Deficiency (GLUT1-DS). Common Anti-Epileptic Drugs (AEDs): Levetiracetam, Diazepam, Phenobarbital, Fenitoin, Clonazepam, Midazolam; Not available (n.a.): Not done (/)

Seven patients were male and six female. Regarding the diagnosis, 15.4% were affected by Pyridoxine-dependent epilepsy (PDE), 23.1% by Molybdenum-Cofactor Deficiency (MCD), 15.4% by infantile Neuronal Ceroid Lipofuscinosis (CLN10), 30.8% by hypophosphatasia (HYPOPH), and finally 15.4% were affected by GLUT1-Deficiency Syndrome (GLUT1-DS).

In all the described cases, seizures started in the first week of life and the mean age of onset was 3.5 days and in all of them, an antiepileptic treatment was started in the first week of life. The EEG, intercritical or video EEG, was pathological in 100% of patients (Table 1).

In 69.2% of the collected cases, the EEG pattern was characterized by burst suppression. In the remaining cases, intermittent paroxysms of sharp waves, excessive multifocal sharp wave discharges, discontinuity for the stated postconceptual age were described (Table 1).

In ten patients, only an intercritical EEG was performed, while three patients underwent a full video-EEG record. The clinical features of seizures were different and could manifest as infantile spasms, tonic, tonic–clonic seizures, myoclonic seizures, low-oxygen saturation, apnea and eye rolling with "arm bicycling"- like movements. In some cases, also hiccups and excessive fetal movements could be considered as an intrauterine form of presentation (Table 1).

Regarding treatment, 76.9% of infants received non-specific treatment with common antiseizure drugs. Five initially had resolution of seizures while other five did not respond to the treatment.

In only three patients, seizures were treated with first-line targeted metabolic treatment, before the occurrence of drug resistance. Among them, one did not respond while two had resolution of seizures.

The evaluation of the neurological outcome showed a developmental delay in 53.8% of patients while it was normal in 38.5%. At follow-up all patients except one were evaluated, but seven of 13 patients died.

## Discussion

Inborn errors of metabolism (IEM) are a rare cause of epilepsy, even if seizures and epilepsy are frequently present in patients with IEM. The diagnosis of those Inborn Error of Metabolism (IEMs) not included in the ENS with tandem mass can be a real challenge for clinicians but seems to have a key-role for therapeutic purposes, in particular for the risk of recurrence and for the neurological outcome.

The aim of our revision of the literature was to investigate those rare IEMs not included in the ENS (e.g. hypophosphatasia, Molybdenum-Cofactor Deficiency, GLUT1-Deficiency Syndrome).

We found that seizures have an early onset; in fact, in all patients, they started in the first week of life. Seizures can present themselves clinically in a very different way. It is remarkable that it’s uncommon to have NS as isolated presentation of IEMs, rather in this patients NS present usually in variable combinations with other clinical and neurological manifestations that should be investigated.

Physical examination can reveal typical features of IEMs such as microcephaly or plagiocephaly (PDE, MCD, CLN10); dysmorphisms: enophthalmos, narrow bifrontal diameter (MCD); muscle tone alterations, especially hypotonia; cried with a high-pitched voice; signs of rickets: craniotabes, rachitic rosary, scoliosis, and Harrison-groove thoracic deformity (HP). In seven patients, major neurological signs such as microcephalia, axial hypotonia with peripheral hypertonia and hyperreflexia, motor delay, and intellectual disability were found. Five patients showed feeding problems as indirect signs of neurological involvement. In our study 12 of 13 patients presented more or less severe neurological alterations, while only one patient did not present any apparent impairment of the Nervous System.

From our systematic review it also emerged that until now there is not a clear diagnostic protocol to follow to detect these rare but insidious IEMs, not usually screened with tandem mass.

For this reason, in this study we have included a flow-chart summarizing the diagnostic process we usually performed in our NICU to make a correct and timely diagnosis of these diseases (Table 2).

**Table 2:**
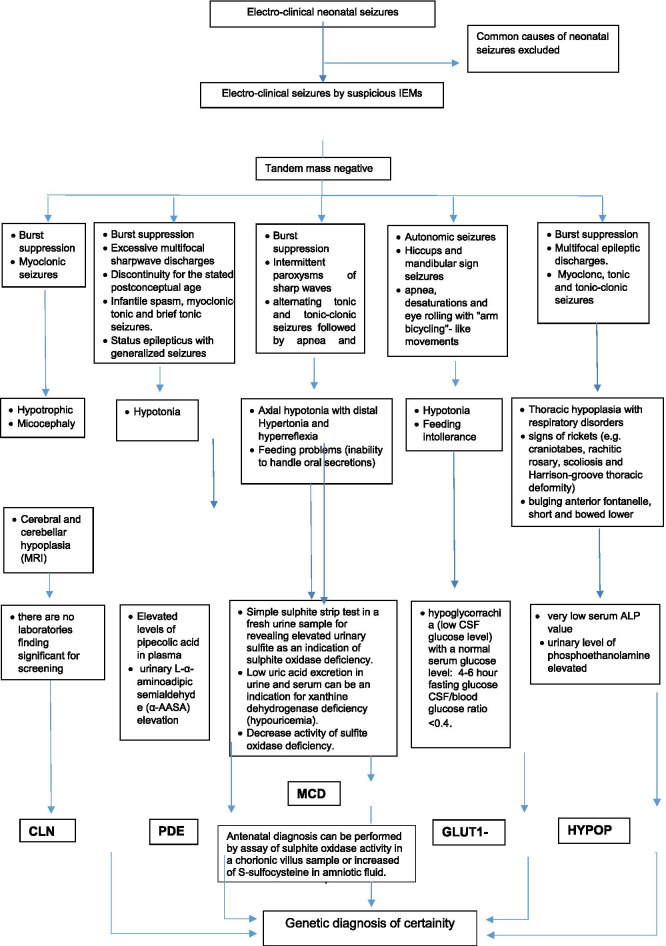
Summary table with the main laboratory tests to make a correct and timely diagnosis of IEMs PDE: Pyridoxine-dependent epilepsy; MCD: Molybdenum-Cofactor Deficiency, CLN10: Infantile Neuronal Ceroid Lipofuscinosis; HYPOPH: hypophosphatasia; GLUT1-DS: GLUT1-Deficiency; MRI: Magnetic Resonance Imaging; ALP: Alkaline Phosphatase; CSF: Cerebrospinal Fluid; IEMs: Inborn Errors of Metabolism

In our patients, EEG did not show a specific pattern for each IEMs; nevertheless, suppression burst was the most frequent findings. Also brain MRI is not ever diagnostic but in some cases could be suggestive of a metabolic disorder (e.g. cerebral and cerebellar atrophy, thin corpus callosum, pachygyria, enlargement of ventricles).

Ten out of 13 infants were treated with common antiseizure drugs (phenobarbital, phenytoin, levetiracetam, clonazepam, vigabatrin, diazepam, midazolam). Among these, five responded to the therapy, showing at first resolution of seizures.

This data, which may seem apparently encouraging, on the contrary caused a delay in the diagnosis of the withstanding metabolic disease responsible for seizures, leading to important consequences in the neurological outcome of these newborns.

In this regard, in all these five infants who responded, at first, to the common antiseizure drug therapy, alterations such as intellectual disability, motor delay up to severe neurological abnormalities were found at the follow-up.

In these infants, therefore, seizures occurred in the following months, leading to a thorough diagnostic investigation that revealed IEMs as their original aetiology. A target therapy was then started with B6 vitamin (os or i.v.) at maximum dosage, or with a ketogenic diet showing a total resolution of seizures (Falsaperla et al. 2020). Nevertheless, their neurological development was already compromised.

In the other five cases resistant to antiepileptic therapy, more in-depth investigations were necessary, making it possible to reach an early diagnosis and therefore an early start of the specific therapy. In these newborns, the neurological outcome was normal in 60% of cases.

Among the studied patients, 23% received specific therapy for the disease as their first treatment (PDE: B6 vitamin at a dosage of 100 mg once daily os; HPH: B6 vitamin at the following dosage: 40 mg/kg/day i.v.)(Gospe 2010)(Pietz et al. 1993).

In all of these five cases, we observed a total resolution of seizures and a good neurological outcome. Only one patient showed mental retardation. In the latter case, the starting dosage of B6 vitamin was below the therapeutic value, then increased after one month of therapy (Yeghiazaryan et al. 2012).

## Strengths and limits

Strengths of this review are the broad search strategy, the reporting data according to PRISMA and the result of “high quality review” assessed using the “Assessing the Methodological Quality of Systematic Reviews 2” (AMSTAR 2) criteria.

Nevertheless, some limitations may have influenced our study selection and results. Only case report in literature were available to meet our inclusion criteria about seizures caused by IEMs not screened with tandem mass. Numerous studies were excluded because the seizures were only clinical and not EEG proven. Finally, language bias may have occurred by excluding non-English articles.

## Conclusion

Neonatal seizures could be included among the first clinical signs of IEMs. Considering the underlying metabolic derangement, NS due to IEMs not diagnosed with tandem mass can partially respond to conventional treatments delaying the diagnosis of IEM with important consequences on the neurological development of the infant.

The current guidelines also indicate the start of treatment with B6 vitamin or ketogenic diet only after evidence of drug resistance, causing an increase of this delay.

Therefore, it is essential to suspect this metabolic form, to pay attention to the first- and second-line tests and to promptly start the appropriate diagnostic investigations. It is also important to introduce trials with pyridoxine, pyridoxal phosphate and Ketogenic diet, before the onset of drug resistance, with a prompt diagnostic work-up, since the diagnosis in these cases is primarily based on a clinical suspicion. This would reduce neurological impairment.

Prompt diagnosis and the onset of appropriate therapy may improve prognosis, neurological outcome and in some cases avoid death; also in cases where a specific therapy is not available, diagnosis will be crucial for family counselling.

## Data Availability

The datasets generated during and/or analysed during the current study are available from the corresponding author on reasonable request.
